# LncRNA Landscape of Coronary Atherosclerosis Reveals Differentially Expressed LncRNAs in Proliferation and Migration of Coronary Artery Smooth Muscle Cells

**DOI:** 10.3389/fcell.2021.656636

**Published:** 2021-05-18

**Authors:** Yaqing Zhou, Sheng Zhang, Wenfeng Ji, Xiongkang Gan, Lei Hua, Can Hou, Jiaxin Chen, Yanjun Wang, Shu He, Hanxiao Zhou, Enzhi Jia

**Affiliations:** Department of Cardiovascular Medicine, The First Affiliated Hospital of Nanjing Medical University, Nanjing, China

**Keywords:** coronary heart disease, coronary artery, RNA sequencing, long non-coding RNA, smooth muscle cell

## Abstract

We aimed to investigate differentially expressed long non-coding RNAs (lncRNAs) and messenger RNAs (mRNAs) in atherosclerosis and validate the expression of lncRNAs and co-expressed target genes in proliferation and migration models of human coronary artery smooth muscle cells (HCASMCs). Ten coronary artery specimens from a subject who died from a heart attack were employed. The pathological analysis was analyzed by hematoxylin and eosin (H&E) staining, and the lncRNAs and mRNAs were identified by RNA sequencing. Bioinformatic analyses were performed to predict possible mechanisms. The proliferation and migration of HCASMCs were induced with oxidized low-density lipoprotein (ox-LDL). Differentially expressed lncRNAs and mRNAs were validated by quantitative real-time polymerase chain reaction (qRT-PCR). In this study, 68 lncRNAs and 222 mRNAs were identified differentially expressed in atherosclerosis. Gene Ontology (GO) and Kyoto Encyclopedia of Genes and Genomes (KEGG) enrichment analyses showed that the Fanconi anemia pathway may be involved in atherosclerosis. *GON4L* was found to be the co-localized target gene of LNC_000439, and 14 genes had high correlations with the expression of seven lncRNAs. In addition, nine lncRNA–miRNA–mRNA networks were constructed, and 53 co-expressed gene modules were detected with weighted gene co-expression network analysis (WGCNA). LNC_000684, LNC_001046, LNC_001333, LNC_001538, and LNC_002115 were downregulated, while LNC_002936 was upregulated in proliferation and migration models of HCASMCs. In total, six co-expressed mRNAs were upregulated in HCASMCs. This study suggests that the differentially expressed lncRNAs identified by RNA sequencing and validated in smooth muscle cells may be a target for regulating HCASMC proliferation and migration in atherosclerosis, which will provide a new diagnostic basis and therapeutic target for the treatment of cardiovascular diseases.

## Introduction

Coronary heart disease (CHD), also known as coronary artery disease (CAD), is the leading cause of morbidity and mortality globally (Benjamin et al., 2018; Amrein et al., 2020). Traditional risk factors for CAD include diabetes, hyperlipidemia, hypertension, obesity, smoking, and family history. Low-density lipoprotein cholesterol (LDL-C) has been recognized as one of the major risk factors for CAD, which typically develops because of atherosclerotic plaque buildup in the coronary arteries (Case and Waksman, 2020; Jin et al., 2020). According to recent guidelines, it is highly recommended for patients with very high risk to achieve lower levels of LDL-C (Grundy et al., 2019; Mach et al., 2020).

In the pathological processes of atherosclerosis, cholesterol containing oxidized low-density lipoprotein (ox-LDL) accumulates in the arterial wall, which promotes the proliferation and migration of medial vascular smooth muscle cells (VSMCs) in the intima and the formation of foam cells (Orekhov et al., 2020). Aberrant proliferation and migration of VSMCs lead to vascular remodeling during vasculopathy and atherosclerosis (Zhang et al., 2020). Furthermore, VSMCs are exposed to a complex microenvironment, resulting in the mutability of VSMCs. Therefore, the differentially expressed genes and molecular mechanisms of VSMCs participating in atherosclerosis are potential therapeutic targets.

Long non-coding RNAs are over 200 bp single-stranded RNA molecules without protein-coding potential that can function as molecular signals, decoys, guides, scaffolds, or enhancers to regulate gene expression and functions (Robinson et al., 2020). Zhou et al. reported that lncRNA CRNDE regulates the proliferation and migration of VSMCs (Zhou Y. et al., 2019). LncRNA GAS5 was found to regulate vascular smooth muscle cell cycle arrest and apoptosis *via* the p53 pathway (Tang et al., 2019). The phenotypic switch of VSMCs was suppressed by lncRNA CASC2 to inhibit vascular remodeling (Gong et al., 2019). Thus, the vital role of lncRNAs in the pathological processes of atherosclerosis and VSMCs is increasingly important.

In this research, we conducted RNA sequencing analysis to identify differentially expressed lncRNAs and mRNAs of varying degrees of coronary atherosclerosis. Then, proliferation and migration models of human coronary artery smooth muscle cells (HCASMCs) were established with ox-LDL, and the relative expression of differentially expressed lncRNAs was validated using qRT-PCR. This work might provide a novel insight into ox-LDL-induced HCASMC dysfunction in atherosclerosis and may suggest a promising strategy for CAD.

## Materials and Methods

### Study Subjects, Coronary Artery Segment Preparation, Pathological Analysis, and RNA Sequencing

The methods and materials have been described in our previous study (Pan et al., 2019).

### Bioinformatic Analysis

Differentially expressed lncRNAs and mRNAs were identified using the R Limma package (version 3.45.9). Kyoto Encyclopedia of Genes and Genomes (KEGG) and Gene Ontology (GO) functional enrichment analyses of these lncRNAs were performed using the clusterprofiler package in R (version 3.17.0). *Cis* target gene prediction involved the identification of mRNA genes located within 100,000 bp upstream or downstream of the lncRNAs as the target genes of the lncRNA. To perform weighted gene coexpression network analysis (WGCNA), we used the R WGCNA package (version 1.69) to build gene coexpression networks. Three databases, miRDB^1^, TargetScan^2^), and DIANA-TarBase^3^, were used to predict the competing endogenous RNA (ceRNA) network of lncRNA–miRNA–mRNA, and the intersection of the results was analyzed.

### Human Coronary Artery Smooth Muscle Cell Culture and Stimulation

Human coronary artery smooth muscle cells were purchased from Sigma. HCASMCs were cultured in a humidified incubator at 37°C supplied with 5% CO_2_ using Dulbecco’s modified Eagle’s medium (DMEM; Gibco, United States) supplemented with 10% fetal bovine serum (FBS; Gibco) and 1% penicillin (100 μ/ml) and streptomycin (100 μg/ml) (Beyotime, Shanghai, China). The cells were divided into two groups. One was cultured with basic DMEM, and the other was administered 0, 25, 50, 75, and 100 mg/L ox-LDL purchased from Yiyuan Biotechnologies (Guangzhou, China) to simulate an abnormal lipid environment and build proliferation and migration models.

### Cell Counting Kit 8 Analysis

For the cell viability and proliferation assay, Cell Counting Kit 8 (CCK-8) was conducted using a CCK-8 assay kit (APExBIO, United States). HCASMCs (5,000 cells/well) in 100 μl were plated in a 96-well microplate (Corning, NY, United States). The absorbance was measured at 450 nm every 12 h using a microplate reader (Multiskan FC, Thermo Fisher Scientific, Waltham, MA, United States) according to the manufacturer’s instructions.

### Cell Cycle Analysis

Two groups of cells were treated with basic DMEM and ox-LDL (50 mg/L) for 24 h. HCASMCs (1 × 106) were detached from six-well plates using 0.25% trypsin–ethylene diamine tetraacetic acid (EDTA) (Beyotime, Shanghai, China) (Corning, NY, United States). Digested cells were collected and washed with phosphate buffered saline (PBS; Beyotime, Shanghai, China), centrifuged twice (2,000 × *g* rpm, 5 min), fixed in 70% ethanol, and kept at 4°C overnight. Next, the cells were centrifuged at 1,000 × *g* rpm for 3 min and washed with PBS to remove residual ethanol. The centrifugation pellet was then resuspended in 500 μl of working fluid containing 10% RNase A and 90% propidium iodide (KeyGen BioTECH, Nanjing, China) for 60 min in the dark. The red fluorescence of propidium iodide was analyzed at 488 nm. Cell cycle analysis was conducted with a flow cytometer (Gallios Flow Cytometer; Beckman Coulter, United States), and data were processed with the corresponding software (Kaluza for Gallios; Beckman Coulter, United States).

### Wound Healing Assay

Migration of HCASMCs was tested by a wound healing assay. Cells at 95% confluence were plated on six-well plates (Corning, NY, United States) in a monolayer. A sterile 200-μl pipette tip was used to scratch across the monolayer of cells. After 24 h, cells were photographed using an Olympus-CKX53 microscope (Olympus, Tokyo, Japan). The wound area was measured by ImageJ software (version 1.46, NIH, United States). The cell migration rate was calculated as follows: cell migration rate = (wound area at 0 h–wound area at 24 h)/wound area at 0 h × 100%.

### Transwell Assay

HCASMCs were plated on six-well plates and stimulated with 50 mg/L ox-LDL for 24 h. Then, 200 μl cells (1 × 10^5^ cells/ml) were seeded into the upper compartment of Transwell chambers (3422, Corning, New York, NY, United States). Then, 500 μl DMEM with 10% FBS was added to the lower chambers, and all of the Transwell chambers were incubated at 37°C in a 5% CO_2_ atmosphere for 24 h. After removing the medium, the cells on the upper side of the filter membrane were removed using a cotton swab, and all of the cells that migrated to the lower side were fixed in 4% glutaraldehyde for 30 min. Finally, the fixed cells were stained with 0.1% crystal violet for 30 min and washed with PBS, and then the migratory cells were counted and photographed with an Olympus-CKX53 microscope.

### qRT-PCR

Total RNA was extracted using TRIzol (Invitrogen), and cDNA was prepared with TransScript One-Step gDNA Removal (Vazyme, Nanjing, China) and cDNA Synthesis SuperMix (Vazyme) according to the manufacturer’s instructions. cDNA was amplified in StepOnePlus (Applied Biosystems) equipment with SYBR qPCR Master Mix (Vazyme). The oligonucleotide sequences of the primers used for qRT-PCR are shown in Table 1. glyceraldehyde-3-phosphate dehydrogenase (GAPDH) was used as an internal reference gene to standardize cycle threshold (CT) values to estimate gene expression. Relative lncRNA expression was calculated using the 2^–ΔΔ*ct*^ method. Each sample and gene were processed in three parallel holes to ensure quantitative accuracy.

**TABLE 1:** Summary of the oligonucleotide primer sequences.

**Gene/LncRNA**	**Forward primer**	**Reverse primer**
GAPDH	GTCTCCTCTGACTTCAACAGCG	ACCACCCTGTTGCTGTAGCCAA
LNC_000684	CTGATGCTCCCAACTGTCTACC	CCAACGTGCCCATTTCTTT
LNC_001046	GCTTCTCAACAGCGAAACAA	GGTGGCTCAGCTCCAATCT
LNC_001333	CTTTGAGCCTTTCCTCTGCC	ACCTCCCCGAAGTCTCCTAC
LNC_001538	ATTCTTGGTGAGGTCTTTTGC	CCTGGATTTTACCCCTGTTCT
LNC_002115	ATGTGGGAGATGGGGAAGGC	ATCGGCAAGGTTTGGCTGAC
LNC_002936	GGACCTCATTCCCAAGCAT	GGGTGGCAGCAGGAGTAAA
STMN1	CCTTATCCCAGTTGATTGTGCAGA	TTTGACCGAGGGCTGAGAATC
SNX10	TTCAGAAAGTTCATCCTCTGGGC	GATTCCTGCGGAGCTGTATTTAC
SCAND1	ACGGTGTGTTGTACTTTGTCATCG	AAGTCCTCTCGCTTGGTCAAAC
MSRA	GGAACTGCTCAAGGTCTTCTGG	CTCCATTTGCTTGGCAGAGGTC
M6PR	ACTTGCGACTTGGTAGGAGAAAAG	GGCACACCCTGAAGATGTAGATG
QRICH1	CACCAGTGTTCAGCCACAAACC	GGTTGCTCAGTAGGCTGGTGAA

*GAPDH, glyceraldehyde-3-phosphate dehydrogenase; STMN1, stathmin 1; SNX10, sorting nexin 10; SCAND1, SCAN domain containing 1; MSRA, methionine sulfoxide reductase A; M6PR, mannose-6-phosphate receptor; QRICH1, glutamine rich 1.*

### Data Analysis

Statistical analyses were performed with SPSS (version 25.0), GraphPad Prism (version 8.0), and R software (version 3.5.1). Data normality and homogeneity of variance were calculated using SPSS 25.0 software. Statistical analyses were then performed with *t*-tests (parametric unpaired or paired, two groups of analysis) and Mann–Whitney *U* tests (non-parametric unpaired). Multiple-group analysis was performed by ANOVA. Data are represented as the mean ± SD unless otherwise stated. When a *p* value was less than 0.05, the result was considered to be of statistical significance. All experimental data were presented using GraphPad Prism 8.0 software and R 3.5.1 software.

## Results

### Global Analysis of Coronary Artery RNA Sequencing Identified lncRNAs and mRNAs

**FIGURE 1:**
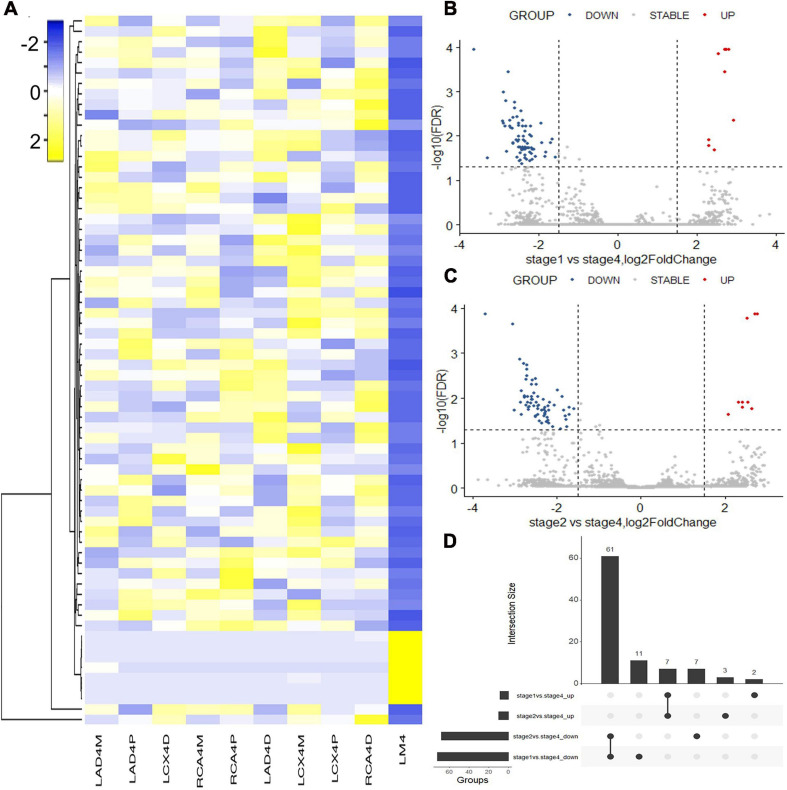
Differentially expressed lncRNAs identified with RNA sequencing. **(A)** Heatmap of the differentially expressed lncRNAs between coronary artery segments. **(B)** Volcano map of differentially expressed lncRNAs between stage 1 and stage 4. **(C)** Volcano map of differentially expressed lncRNAs between stage 2 and stage 4. **(D)** The intersection of differentially expressed lncRNAs in stages 1 and 2 vs. stage 4. Stage: 0, normal tunica intima; 1, fatty streak tunica intima; 2, fibrous plaques tunica intima; 3, atherosclerotic tunica intima; 4, secondary affection tunica intima.

**FIGURE 2:**
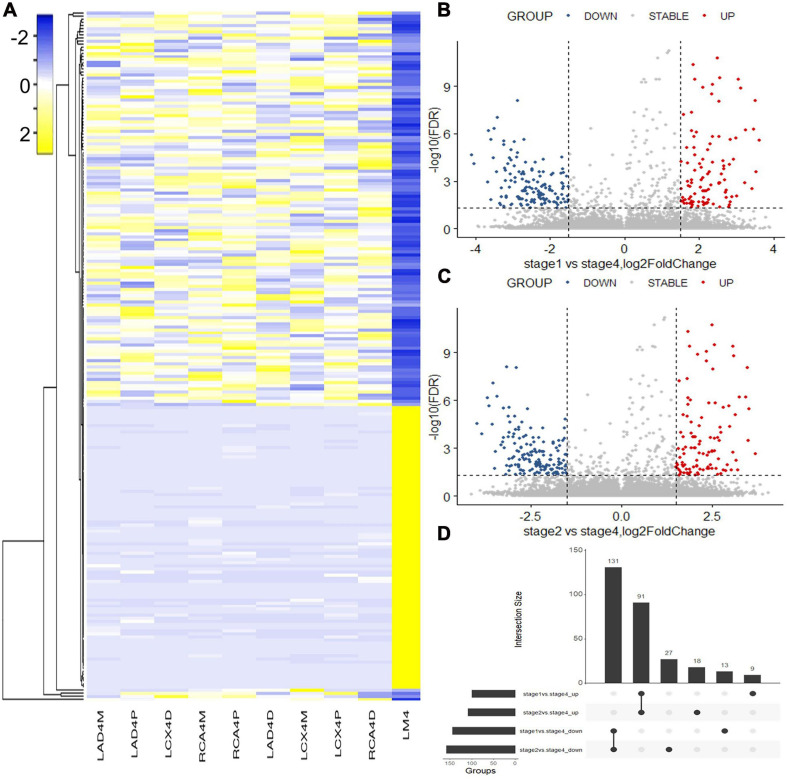
Differentially expressed mRNAs identified with RNA sequencing. **(A)** Heatmap of the differentially expressed mRNAs between coronary artery segments. **(B)** Volcano map of differentially expressed mRNAs between stage 1 and stage 4. **(C)** Volcano map of differentially expressed mRNAs between stage 2 and stage 4. **(D)** The intersection of differentially expressed mRNAs in stages 1 and 2 vs. stage 4. Stage: 0, normal tunica intima; 1, fatty streak tunica intima; 2, fibrous plaques tunica intima; 3, atherosclerotic tunica intima; 4, secondary affection tunica intima.

Hematoxylin and eosin (H&E) staining was used to evaluate the natural history and histological classification of coronary atherosclerotic lesions, and the results of H&E staining of histologic characterization were shown in our previous study (Pan et al., 2019). RNA sequencing of coronary artery segments was performed to detect the differentially expressed lncRNAs and mRNAs. After normalization, all the data in these segments are presented in the heatmap (Figures 1A, 2A). A volcano plot showed the highly or lowly expressed (fold change > 2, *p* < 0.05) lncRNAs (Figures 1B,C) and mRNAs (Figures 2B,C) of stage 1 and stage 2 vs. stage 4 in the data. Taking the intersection, 68 (seven upregulated and 61 downregulated) lncRNAs and 222 (91 upregulated and 131 downregulated) mRNAs were included (Table 2; Figures 1D, 2D).

**TABLE 2:** Summary of the comparisons of lncRNA and mRNA expression levels in different stages of atherosclerosis.

**Differential analysis**	**Intersection**	**Stage 1 vs. Stage 4**	**Stage 2 vs. Stage 4**
Differentially expressed lncRNAs	68	81	78
Upregulated lncRNAs	7	9	10
Downregulated lncRNAs	61	72	68
Differentially expressed mRNAs	222	244	267
Upregulated mRNAs	91	100	109
Downregulated mRNAs	131	144	158

*Stage: 0, normal tunica intima; 1, fatty streak tunica intima; 2, fibrous plaques tunica intima; 3, atherosclerotic tunica intima; 4, secondary affection tunica intima.*

### Gene Ontology and Kyoto Encyclopedia of Genes and Genomes Enrichment of the Differentially Expressed Genes

**FIGURE 3:**
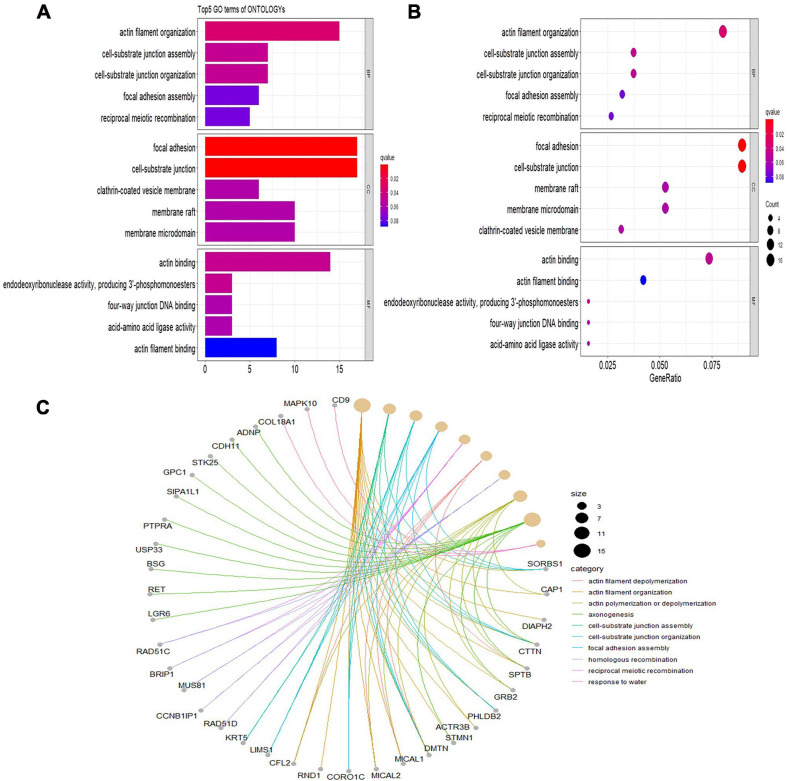
Significantly enriched Gene Ontology (GO) terms of differentially expressed genes based on their functions. **(A)** Bar chart of the top 5 BP, CC, and MF terms in the enrichment analysis. **(B)** Bubble map of the top 5 BP, CC, and MF terms in the enrichment analysis. **(C)** Chord plot of the top 5 BP, CC, and MF terms in the enrichment analysis. BP, biological process; CC, cellular component; MF, molecular function.

**FIGURE 4:**
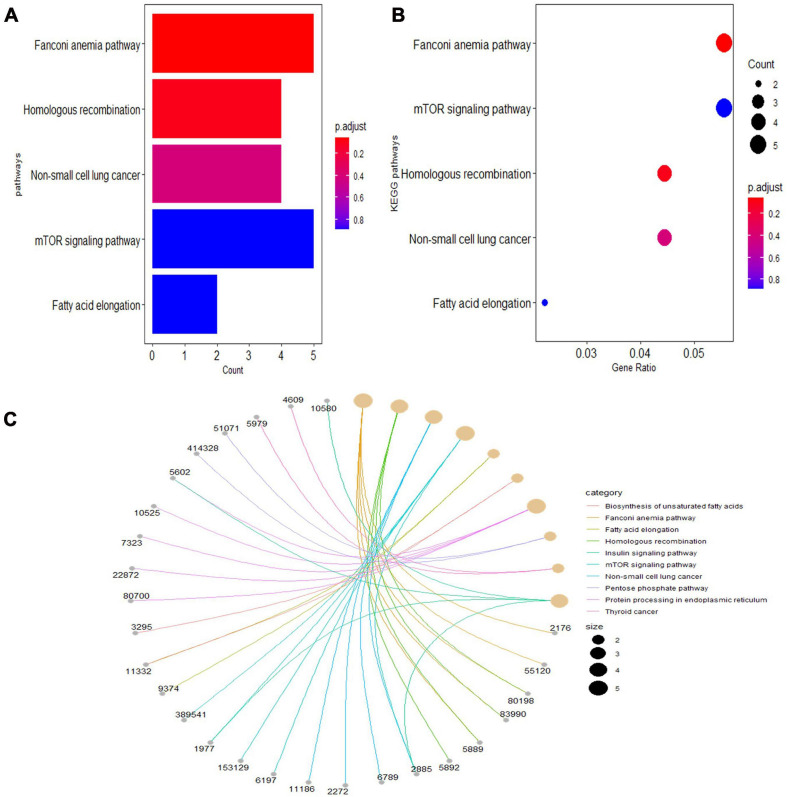
The top 5 enriched Kyoto Encyclopedia of Genes and Genomes (KEGG) pathway terms of differentially expressed genes. **(A)** Bar chart of the top 5 KEGG pathways in the enrichment analysis. **(B)** Bubble map of the top 5 KEGG pathways in the enrichment analysis. **(C)** Chord plot of the top 5 KEGG pathways in the enrichment analysis.

Gene Ontology analyses were performed on three different aspects, including biological process (BP), cellular component (CC), and molecular function (MF), reflecting the dynamic alteration processes during different stages of atherosclerosis (Figure 3). According to the functional enrichment results, 78 BP terms, 129 CC terms, and 47 MF terms were enriched in differentially expressed genes (DEGs). The most enriched BP terms included actin filament organization, cell–substrate junction assembly, and cell–substrate junction organization. Focal adhesion, cell-substrate junctions, and clathrin-coated vesicle membranes were the most enriched CC terms. The most enriched MF terms were actin binding, endodeoxyribonuclease activity, producing 3′-phosphomonoesters, and four-way junction DNA binding. Next, a KEGG pathway enrichment analysis was performed to analyze the most significantly enriched pathways for DEGs (Figure 4). A total of 178 pathways were enriched in DEGs, and the top 5 enriched pathways were the Fanconi anemia pathway, mechanistic target of rapamycin kinase (mTOR) signaling pathway, homologous recombination, non-small-cell lung cancer, and fatty acid elongation pathway.

### Target Genes of lncRNAs and ceRNA Interaction Model

**FIGURE 5:**
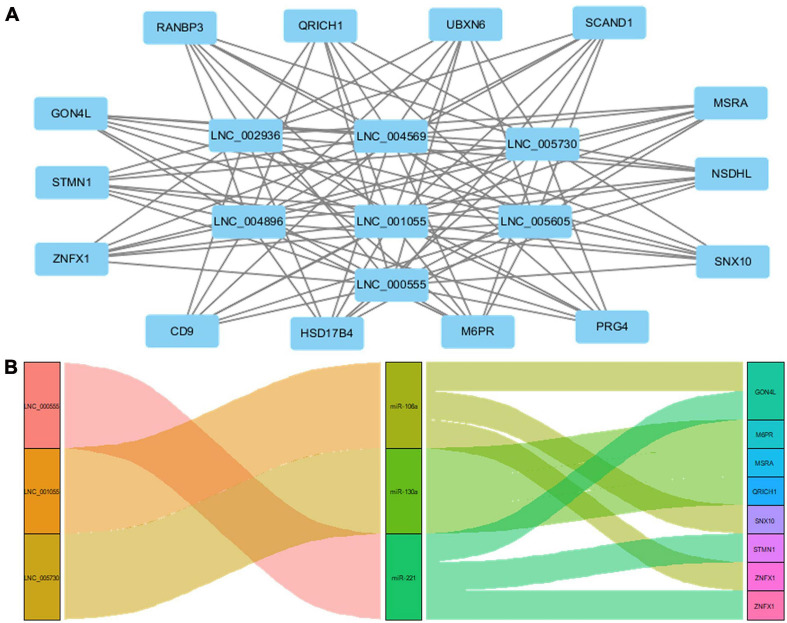
Target genes of lncRNAs and ceRNA interaction model. **(A)** The network of coexpressed target genes of lncRNAs. **(B)** The network of lncRNA–miRNA–mRNA interaction model.

*Cis* function prediction analysis of the 68 lncRNAs and 222 mRNAs based on positional relations showed that *GON4L*, which is located 72,001 upstream, is the co-localized target gene of LNC_000439. Pearson correlation was applied to analyze the correlation between differentially expressed lncRNAs and mRNA expression in coronary artery samples. The results showed that *GON4L, STMN1, ZNFX1, CD9, HSD17B4, M6PR, PRG4, SNX10, NSDHL, MSRA, SCAND1, UBXN6, QRICH1*, and *RANBP3* are co-expressed target genes of LNC_002936, LNC_004569, LNC_005730, LNC_004896, LNC_001055, LNC_005605, and LNC_000555. The Pearson correlations of the top 3 correlated genes are presented in Table 3, and the regulatory network of lncRNAs and co-expressed genes is shown in Figure 5A. Then, using the following three databases, including miRDB, TargetScan, and DIANA-TarBase, as described in the “Materials and Methods” section, and intersected with the RNA sequencing results to predict lncRNA–miRNA–mRNA (ceRNA) networks, LNC_000555, LNC_001055, and LNC_005730 can affect the expression of *GON4L, M6PR, MSRA, QRICH1, SNX10, STMN1*, and *ZNFX1* by adsorbing miRNAs, including miR-106a, miR-130a, and miR-221 (Figure 5B).

**TABLE 3:** Summary of the top 3 correlated coexpressed genes of lncRNAs.

**LncRNA**	**mRNA gene symbol**	**Pearson correlation**	***p* value**
LNC_002936	CD9	0.99966204	5.7 × 10^–14^
	NSDHL	0.99958603	1.3 × 10^–13^
	ZNFX1	0.99951061	2.5 × 10^–13^
LNC_004569	GON4L	0.99341329	8.2 × 10^–9^
	MSRA	0.99298806	1.1 × 10^–8^
	STMN1	0.99226337	1.6 × 10^–8^
LNC_005730	SNX10	0.99862558	1.6 × 10^–11^
	SCAND1	0.99825014	4.1 × 10^–11^
	PRG4	0.99815934	5.0 × 10^–11^
LNC_004896	GON4L	0.99976957	1.2 × 10^–14^
	STMN1	0.99972796	2.4 × 10^–14^
	UBXN6	0.99967951	4.7 × 10^–14^
LNC_001055	ZNFX1	0.99993876	6.2 × 10^–17^
	UBXN6	0.99993671	6.5 × 10^–17^
	STMN1	0.99993634	7.2 × 10^–17^
LNC_005605	UBXN6	0.99904520	3.8 × 10^–12^
	ZNFX1	0.99903404	3.8 × 10^–12^
	PRG4	0.99902301	4.0 × 10^–12^
LNC_000555	GON4L	0.99980242	6.7 × 10^–15^
	STMN1	0.99979437	7.8 × 10^–15^
	UBXN6	0.99975221	1.7 × 10^–14^

*CD9, CD9 molecule; NSDHL, NAD(P) dependent steroid dehydrogenase-like; ZNFX1, zinc finger NFX1-type containing 1; GON4L, gon-4 like; MSRA, methionine sulfoxidereductase A; STMN1, stathmin 1; SNX10, sorting nexin 10; SCAND1, SCAN domain containing 1; PRG4, proteoglycan 4; UBXN6, UBX domain protein 6; ZNFX1, zinc finger NFX1-type containing 1.*

### Weighted Gene Co-expression Network Analysis

**FIGURE 6:**
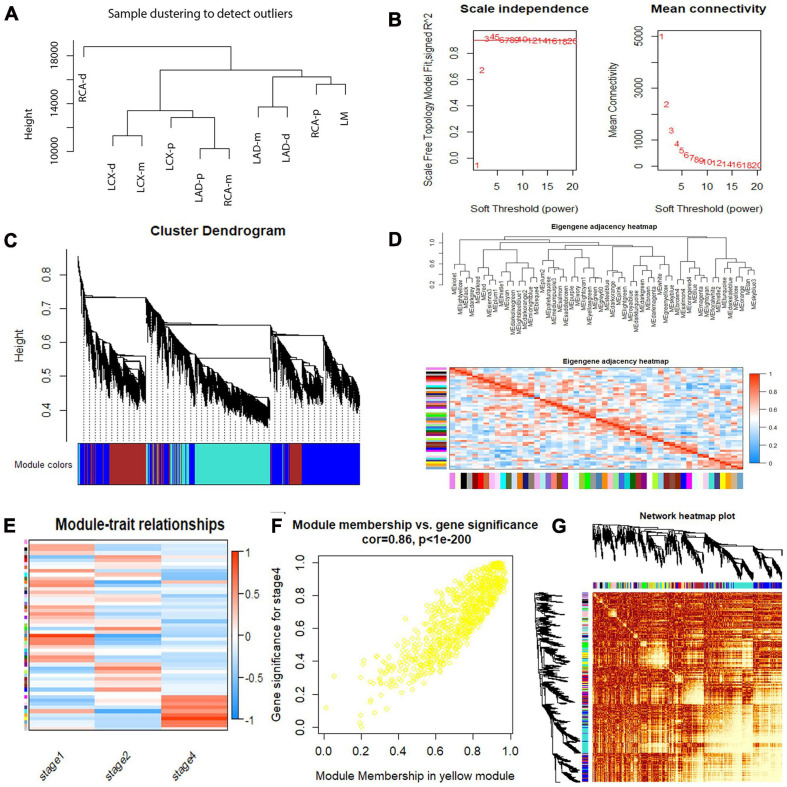
Weighted gene coexpression network analysis (WGCNA) summary. **(A)** Sample clustering to detect outliers. **(B)** Analysis of the scale-free fit index and the mean connectivity for various soft-thresholding powers. **(C)** Cluster dendrogram based on the dissimilarity of the topological overlap matrix. **(D)** The branches of the dendrogram and the eigengene adjacency heatmap. **(E)** Module–trait relationship. **(F)** A scatterplot of gene significance (GS) vs. module membership (MM) in the yellow module. A highly significant correlation between GS and MM in the yellow module (correlation = 0.86; *P* value < 1^*e*−200^) was detected. **(G)** Network heatmap of the 53 modules.

To identify co-regulated sets of genes, also known as modules participating in the initiation and development of atherosclerosis, we performed a WGCNA of our RNA sequencing data set for atherosclerosis at different developmental stages. A total of 15,501 genes were included, and 53 coexpressed gene modules were detected. The module size ranged from 53 to 2,967 genes. Each module was assigned a different color. Hierarchical clustering of samples to detect outliers used in the analysis is shown in Figure 6A. We chose the power of β = 3 (scale-free R^2^ = 0.9116, slope = −1.154) as the soft thresholding to construct a scale-free network (Figure 6B). Next, a cluster dendrogram was generated according to the dissimilarity of the topological overlap matrix, and the different colors correspond to the coexpression modules in atherosclerosis (Figure 6C). Acceptable discriminability was presented between modules in the similarity heatmap plot (Figure 6D). The module eigengene (ME) is the first principal component of each module and represents the expression levels of all genes in the module. Gene significance (GS) was used to evaluate the correlation between genes and clinical features with linear regression. The module significance (MS) was calculated by averaging the absolute GS values of all genes in the coexpression module. The most statistically significant module (*p* < 0.0001) was further analyzed. By gathering similarly expressed genes, 53 coexpressed gene modules were detected (Figure 6E). The MEs in the yellow module showed a higher correlation with the stage of atherosclerosis (R^2^ = 0.86, *p* = 7.29E-10) (Figure 6F). Then, a network heatmap plot (Figure 6G) was created by WGCNA showing overall module-related gene branches in hierarchical clustering dendrograms.

### Proliferation and Migration of Human Coronary Artery Smooth Muscle Cells Induced by ox-LDL

Oxidized low-density lipoprotein was administered to HCASMCs *in vitro* to simulate abnormal lipid metabolism in atherosclerosis. We performed CCK-8 assay and cell cycle analysis to evaluate the proliferation of HCASMCs induced by ox-LDL at 0, 25, 50, 75, and 100 mg/L for different times. The CCK-8 results indicated that the cell proliferation level was significantly increased in the experimental groups (Supplementary Figure 1A). Cell cycle analysis showed that the cell population increased in the S and S + G2 phases and decreased in the G1 phase, which confirms the result (Supplementary Figure 1B). Wound healing and Transwell assays were performed to validate the migration of HCASMCs exposed to 50 mg/L for 24 h. There was a significant decrease in the wounded area at 24 h post scratch vs. controls (Supplementary Figure 1C). In Transwell assays, the intervention group had more cells that migrated to the lower chamber than the control group (Supplementary Figure 1D). Therefore, the proliferation and migration of HCASMCs can be induced by ox-LDL at 50 mg/L for 24 h.

### Validation of the Differentially Expressed lncRNAs and Coexpressed mRNAs in Human Coronary Artery Smooth Muscle Cells

**FIGURE 7:**
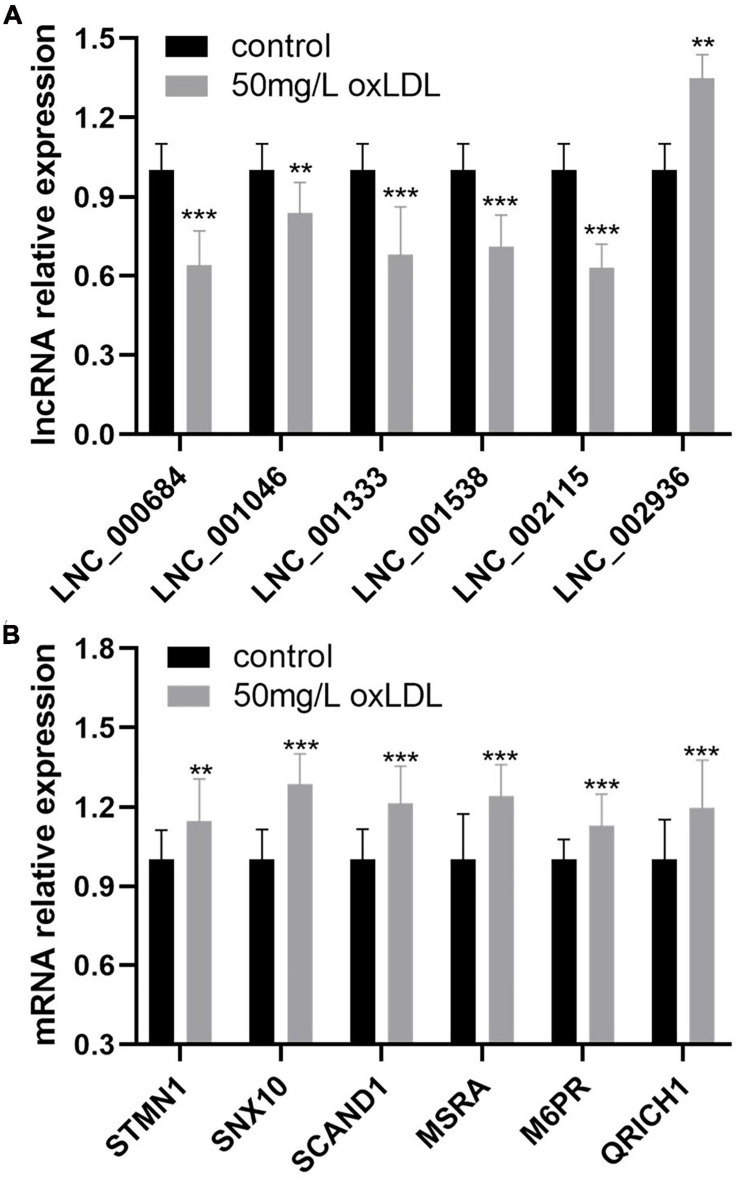
The relative expression of six lncRNAs and coexpressed mRNAs in human coronary artery smooth muscle cells (HCASMCs) induced with oxidized low-density lipoprotein (ox-LDL). **(A)** LNC_000684, LNC_001046, LNC_001333, LNC_001538, and LNC_002115 were downregulated while LNC_002936 was upregulated in the proliferation and migration models of HCASMCs induced with 50 mg/L ox-LDL for 24 h. **(B)** STMN1, SNX10, SCAND1, MSRA, M6PR, and QRICH1 were upregulated in the proliferation and migration models of HCASMCs induced with 50 mg/L ox-LDL for 24 h. **p < 0.01, ***p < 0.001.

The relative expression of 68 differentially expressed lncRNAs between the proliferation and migration model of HCASMCs and the control groups was validated using qRT-PCR. The results showed that LNC_000684, LNC_001046, LNC_001333, LNC_001538, and LNC_002115 were downregulated, while LNC_002936 was upregulated in the experimental groups, which was consistent with the RNA sequencing results (Figure 7A). In addition, the coexpressed mRNAs of lncRNAs were validated too. Among the 14 coexpressed genes, six of which including *STMN1, SNX10, SCAND1, MSRA, M6PR*, and *QRICH1* were confirmed to be upregulated in the proliferation and migration model of HCASMCs (Figure 7B).

## Discussion

Cardiovascular diseases are a significant health burden with an ever-increasing prevalence and remain the leading causes of morbidity and mortality worldwide (Shaito et al., 2020). HCASMC proliferation and migration are the pathological basis of atherosclerosis (Catalano et al., 2020; Maguire and Xiao, 2020). The purpose of the present study was to provide a comprehensive survey of lncRNAs and mRNAs in human coronary arteries and identify differentially expressed lncRNAs and mRNAs and possible regulatory pathways according to the histological classification of atherosclerotic lesions. In this study, we identified 68 lncRNAs and 222 mRNAs that were differentially expressed in the 10 coronary artery segment samples by RNA sequencing. GO and KEGG enrichment analyses of the DEGs showed that actin filaments, cell–substrate junctions, and focal adhesion may underlie the occurrence and development of atherosclerosis. *GON4L* was found to be the colocalized target gene of LNC_000439, and 14 genes had high correlations with the expression of seven lncRNAs. In addition, nine lncRNA–miRNA–mRNA networks were constructed, and 53 coexpressed gene modules were detected with WGCNA. The relative expression of six lncRNAs, including downregulated LNC_000684, LNC_001046, LNC_001333, LNC_001538, and LNC_002115 and upregulated LNC_002936, was validated in HCASMCs induced with ox-LDL that proliferate and migrate in the pathological processes of atherosclerosis. In total, six coexpressed target genes, *STMN1, SNX10, SCAND1, MSRA, M6PR*, and *QRICH1*, were upregulated in HCASMCs.

RNA sequencing has become an indispensable tool for transcriptome-wide analysis of differential gene expression, including non-coding RNAs and differential splicing of mRNAs (Stark et al., 2019). During the study of different cells involved in atherosclerosis, Zhu et al. (2019) performed RNA sequencing to determine miRNA profiles of exosomes derived from control macrophages and nicotine-treated macrophages and found that exosomal miR-21-3p from experimental groups may accelerate the development of atherosclerosis. RNA sequencing was conducted on peripheral blood mononuclear cells (PBMCs) of early onset myocardial infarction (MI) patients, and lncRNA nuclear enriched abundant transcript (NEAT1) was the most highly expressed lncRNA (Gast et al., 2019). In a previous study of specimens of atherosclerosis, the circRNA, miRNA, and mRNA expression profiles of rabbit carotid arteries were analyzed using RNA sequencing, and seven circRNAs were found to be related to atherosclerosis (Zhang et al., 2018). Using RNA sequencing profiling of the aortic intima of lesions, the authors identified a macrophage-specific lncRNA, Macrophage-Associated Atherosclerosis lncRNA Sequence (MAARS), that regulates apoptosis and efferocytosis in atherosclerosis by tethering ELAV like RNA binding protein 1 (ELAVL1) HuR (Simion et al., 2020). Although those RNA sequencing studies of CAD might reflect the pathophysiological changes in CAD, the comprehensive analysis of differentially expressed lncRNAs and mRNAs of human coronary arteries has not been explored with RNA sequencing before. The present study is different from previous studies because it employed the sampling of human coronary artery segments. A large quantity and variety of high-quality lncRNAs and mRNAs were identified. These RNAs were identified from comprehensive coronary artery tree samples, and histological classification of the atherosclerotic stages was determined by H&E staining. Wide-ranging and deep-going bioinformatic analyses, including GO and KEGG enrichment analyses, target gene prediction, ceRNA interaction models, and WGCNA, were performed to predict possible mechanisms. The top pathway identified in the bioinformatic analysis is the Fanconi anemia pathway, which has been found to regulate important cellular processes such as DNA replication, cell cycle control, and DNA damage repair (Nie et al., 2020). Therefore, the Fanconi anemia pathway might regulate the proliferation and apoptosis of coronary endothelial cells and smooth muscle cells that are associated with atherosclerotic pathology (Wolf and Hunziker, 2020). Previous research indicated that lncRNA can *cis-* or trans-regulate the expression of target genes, which is the main regulation mechanism of lncRNA (Zhou G. F. et al., 2019). The coexpressed target genes in our study are on different chromosomes from the correlated lncRNA, which suggest that these lncRNAs trans-regulate the target genes to affect the occurrence and development of atherosclerosis.

Long non-coding RNAs have emerged as significant players in almost every level of gene function and regulation, and a large number of novel lncRNAs have been uncovered (Qian et al., 2019). A large number of lncRNAs were found to be associated with the proliferation and migration of smooth muscle cells and atherosclerosis. The expression of lncRNA H19 was found to be higher in patients with atherosclerosis, and it promotes atherosclerosis by regulating mitogen-activated protein kinase (MAPK) and nuclear factor (NF)-κ B signaling pathways (Pan, 2017). The lncRNA-FA2H-2-MLKL pathway in atherosclerosis, which regulates autophagic flux and inflammation through the mTOR signaling pathway, was identified by Guo et al. (2019). The lncRNA TUG1 was reported to promote the proliferation of VSMCs and atherosclerosis through the miRNA-21/PTEN axis (Li et al., 2018). Previous studies investigated the functional roles of annotated lncRNAs in cell and animal models; however, no research identified novel differentially expressed lncRNAs and mRNAs from human coronary artery tissues and validated the expression of molecules in HCASMCs. LncRNA can act as a sponge for miRNAs to indirectly regulate miRNA downstream target genes, which is defined as the ceRNA regulation model (Han et al., 2020). In our study, through the construction of the ceRNA network, three upregulated lncRNAs including LNC_000555, LNC_001055, and LNC_005730 may act as sponges that absorb miR-106a, miR-130a, and miR-221. Thus, the posttranscriptional inhibition of the three miRNAs on the target genes including *GON4L, M6PR, MSRA, QRICH1, SNX10, STMN1*, and *ZNFX1* was weakened. The target genes are upregulated and affect the occurrence and development of atherosclerosis. CCK-8, cell cycle analysis, Transwell assay, and wound healing assay confirmed the proliferation and migration model of HCASMCs induced with ox-LDL. We identified six novel differentially expressed lncRNAs and six coexpressed target genes from human coronary artery tissues and validated the expression of molecules in HCASMCs. The present study may shed light on the molecular mechanisms underlying the dysfunction of smooth muscle cells in coronary artery atherosclerosis by combining clinical tissues with cells.

There are some limitations in this study. The human coronary artery samples used in the present study were obtained from one case with 10 segments. This sample size was small, and a large-scale study should be conducted in the future to validate the results of this study. In addition, since molecular mechanism and functional experiments were not included in this study, the specific mechanism by which differentially expressed RNAs may participate in the proliferation and migration of HCASMCs, and the development of atherosclerosis was not explored. To increase the potential clinical utility of the identified lncRNAs and mRNAs as CAD biomarkers, the functional roles of the lncRNAs, signaling pathways, and interaction networks identified in the present study should be clarified and validated in further studies.

In the present study, RNA sequencing analysis of coronary arteries identified 68 lncRNAs and 222 mRNAs that were differentially expressed and associated with atherosclerosis. In addition, enrichment analysis, target gene prediction, ceRNA interaction models, and WGCNA were performed to predict possible mechanisms of atherosclerosis. The relative expression of LNC_000684, LNC_001046, LNC_001333, LNC_001538, and LNC_002115 was downregulated, while LNC_002936 was upregulated, which was validated in HCASMCs induced with ox-LDL. In total, six coexpressed target genes were upregulated in the proliferation and migration model of HCASMCs. This study suggests that the differentially expressed lncRNAs may be a target for regulating HCASMC proliferation and migration in atherosclerosis, which will provide a new diagnostic basis and therapeutic target for cardiovascular diseases.

## Data Availability Statement

The datasets presented in this study can be found in online repositories. The names of the repository/repositories and accession number(s) can be found below: NCBI SRA; PRJNA705037.

## Ethics Statement

The methods were performed in accordance with the approved guidelines, and all experimental protocols were approved by the Ethics Committee of Nanjing Medical University and The First Affiliated Hospital of Nanjing Medical University.

## Author Contributions

EJ conceived the study. YZ and SZ drafted the manuscript. YZ, WJ, XG, and LH performed the experiments. CH, JC, and YW analyzed the data. WJ, SH, and HZ edited the manuscript for intellectual content. All authors read and approved the final manuscript and contributed to the article and approved the submitted version.

## Conflict of Interest

The authors declare that the research was conducted in the absence of any commercial or financial relationships that could be construed as a potential conflict of interest.

## Footnotes

1 http://www.mirdb.org/

2 www.targetscan.org

3 http://www.microrna.gr/tarbase

## Supplementary Material

Supplementary Figure 1 Proliferation and migration of HCASMCs were induced by ox-LDL. **(A)** CCK-8 assay showed the proliferative ability and a marked increase in the viability of HCASMCs after stimulation with ox-LDL at 0, 25, 50, 75, and 100 mg/L. ***p* < 0.01, ****p* < 0.001. **(B)** Cell cycle analysis of HCASMCs stimulated with ox-LDL at 50 mg/L for 24 h. **p* < 0.05. **(C)** Wound healing assay showing quantification of the residual wounded area at 24 h postscratch vs. controls (*n* = 5). ****p* < 0.001. **(D)** Transwell assays were also used to examine cell migration, and quantitative statistical analysis of cell migration data is presented as the means ± SDs from five samples. ****p* < 0.001.
