# Supplementary figures and images for: LncRNA Landscape of Coronary Atherosclerosis Reveals Differentially Expressed LncRNAs in Proliferation and Migration of Coronary Artery Smooth Muscle Cells
Source: Front Cell Dev Biol. 2021 May 18;9:656636. doi: 10.3389/fcell.2021.656636 (PMC8168468; doi:10.3389/fcell.2021.656636)

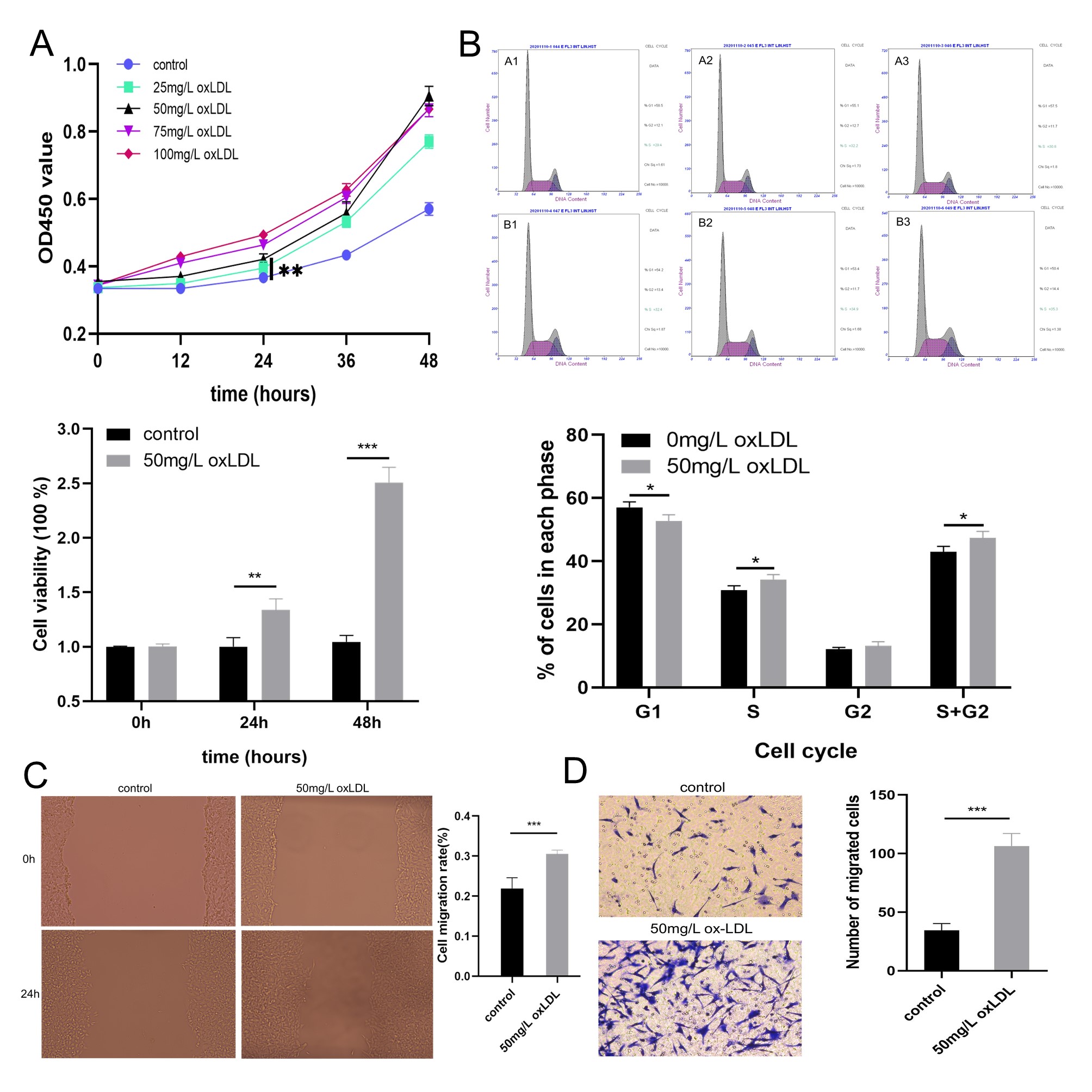

Supplement: Supplementary Figure 1 — Proliferation and migration of HCASMCs were induced by ox-LDL. (A) CCK-8 assay showed the proliferative ability and a marked increase in the viability of HCASMCs after stimulation with ox-LDL at 0, 25, 50, 75, and 100 mg/L. **p < 0.01, ***p < 0.001. (B) Cell cycle analysis of HCASMCs stimulated with ox-LDL at 50 mg/L for 24 h. *p < 0.05. (C) Wound healing assay showing quantification of the residual wounded area at 24 h postscratch vs. controls (n = 5). ***p < 0.001. (D) Transwell assays were also used to examine cell migration, and quantitative statistical analysis of cell migration data is presented as the means ± SDs from five samples. ***p < 0.001. [file Image_1.JPEG]
